# Microstructure and Salt Fog Corrosion of Wrought Mg-Al-Zn and Mg-RE Alloys

**DOI:** 10.3390/ma16031004

**Published:** 2023-01-21

**Authors:** Helen Kamoutsi, Gregory N. Haidemenopoulos, Anette E. Gunnaes, Spyros Diplas

**Affiliations:** 1 Department of Mechanical Engineering, University of Thessaly, 38334 Volos, Greece; 2 Department of Physics, University of Oslo (UiO), 0316 Oslo, Norway; 3 SINTEF Industry, 0314 Oslo, Norway

**Keywords:** magnesium alloys, rare earth elements, corrosion, XPS, weight loss, microstructure, AZ31, AZ61, WE43

## Abstract

Wrought magnesium alloys have received attention due to their potential application as lightweight materials. However, their use is limited by their poor corrosion resistance. Rare earth additions have the potential to enhance corrosion resistance. The present work included a microstructural investigation and corrosion testing of the alloy WE-43, containing Nd and Y, which was compared against the more conventional compositions of AZ31 and AZ61 alloys. All three alloys exhibited a recrystallized equiaxed structure after hot rolling with the presence of second phases—precipitates. The WE-43 alloy exhibited a better corrosion resistance than AZ31 and AZ61 under salt fog testing, indicated by the lower depth of attack and lower weight loss. The second phases in the microstructure of AZ31 and AZ61 alloys determined their corrosion resistance. The second phases in the AZ31 and AZ61 alloys (based on Al-Mg and Al-Mn phases) were nobler than the Mg matrix and catholically acted, thus sacrificing the Mg matrix. The superior corrosion resistance of WE43 was due to the incorporation of Y in the oxide/hydroxide film. In addition, the second phases in the WE43 consisted of Nd and Y and were less noble than the Mg-matrix. Thus, they acted as anodic sites protecting the Mg-matrix. The above results show the beneficial effect of rare earth additions to wrought Mg alloys towards increased corrosion resistance.

## 1. Introduction

Magnesium alloys are considered as potential candidates for many structural applications thanks to their low density [1]. However, in the past, their usage has been limited by their generally poor corrosion resistance and strength at high temperatures.

Magnesium’s commercial use has quickly expanded in response to the desire for light-weighting in transportation and consumer electronics. Mg is now primarily used in cast Mg products, and there is a growing market for wrought Mg products and biomedical Mg components [2]. As a result, there have been several new Mg-alloys reported during the past 20 years.

The opportunities for extruded and sheet magnesium products in the automotive industry, in particular, are increasing as the quest for light-weighting gains momentum. However, the current alloys all have limitations, and these are accentuated when higher productivity targets are also imposed. As the desire for lightweight products increases, so does the demand for extruded and sheet magnesium products in the automotive and aerospace industries. In particular, wrought magnesium alloys, developed in recent years, have increased potential for applications in the transportation sector, provided that the corrosion problem is properly addressed. 

Usually, the corrosion resistance of magnesium alloy is improved by increasing the purity level of the alloy. Magnesium alloys containing yttrium and/or rare earth metals (RE) exhibit an excellent combination of mechanical properties and corrosion resistance. The improved mechanical properties are attributed to solution hardening and precipitation hardening, especially the finely dispersed precipitation of intermetallic particles following the addition of RE [3]. Most of the published literature has concerned the corrosion resistance of cast magnesium alloys. The corrosion behavior of as-cast and solution-treated binary Mg-X alloys was described by Cao et al. [4]. Corrosion was characterized by immersion tests in 3.5% NaCl solution saturated with Mg(OH)_2_, and by salt spray. In both scenarios, the alloys investigated similarly performed and were more severely corroded than high-purity Mg. The faster corrosion rates of the alloys were attributed to the second phase particle in the microstructure that had dissolved. 

The effect of RE on corrosion, focusing on the relevant corrosion mechanisms, has been reported by Birbilis et al. [5]. High-pressure die cast magnesium-rare earth (RE)-based alloys have had their corrosion characteristics examined. Pure Mg was given binary additions of La, Ce, and Nd. Results showed that progressively increasing RE alloying additions increased corrosion rates. This study clarified the microstructure-corrosion relationship by demonstrating the electrochemical impact of precisely controlled binary alloying additions to magnesium. Similar work was performed by Gandel et al. [6], where they studied the effect of Zr addition on the corrosion of Mg. The corrosion rate of Mg was found to be negatively impacted by Zr in both solid solution and metallic particle forms. However, in alloys with several alloying additions, this negative effect was less noticeable. On the other hand, Sudholz et al. [7] studied the effect of Y on the corrosion resistance of cast magnesium-yttrium (Y) alloy and came to similar conclusions.

The mechanism of fracture was investigated for the environment-assisted cracking of WE43-T6 cast alloy by Marrow et al. [8]. Brittle intergranular intermetallic particles acted as initiation sites for cracks, that propagated by transgranular cleavage. The corrosion resistance offered by RE-based conversion coatings on pure magnesium, as well as on WE43 alloy, has been studied by Rudd et al. [9]. The coatings, formed by submersion in solutions containing rare-earth salts, considerably decreased the dissolution of magnesium in a pH 8.5 buffer solution. After extended immersion, the coatings first appeared to become more protective, but, after periods exceeding 60 min, began to deteriorate. This behavior was attributed to the formation of magnesium hydroxy corrosion products and mixed rare earth/magnesium oxide/hydroxide coatings.

The corrosion performance of bare WE43-T6 and AZ91D magnesium alloys and with protection via phosphate conversion coatings has been characterized by Arrabal et al. [10]. Untreated WE43-T6 alloy was initially corroded with the formation of a uniform corrosion layer (Mg(OH)_2_), accompanied by initial pits around magnesium-rare earth intermetallic compounds. Alloy AZ91D exhibited increased corrosion susceptibility, with localized corrosion around β-phase. 

Corrosion products of WE43 commercial alloy have been characterized by various techniques (XPS, ToF-SIMS, SEM, and EDS), as described by Ardelean et al. [11]. Nd-rich precipitates were mainly found at grain boundaries, while Zr/Y-rich zones were distributed inside most of the grains. They came to the following conclusions: neodymium, zirconium, and yttrium play a vital part in the alloy’s marginally increased corrosion resistance, and WE43 exhibits a slower cathodic reaction than AZ91 and pure Mg.

While the corrosion resistance of the cast alloys has been extensively investigated, there is rather limited work on the corrosion of wrought magnesium alloys. Corrosion of extruded AZ61 alloy following salt spray and immersion exposure has been recorded by Martin et al. [12], while the corrosion mechanisms of extruded AZ31 subjected to similar environments has been reported by Walton et al. [13]. Both works revealed that the immersion environment resulted in more prominent general and intergranular corrosion, while the salt-spray environment resulted in more severe pitting corrosion.

The effect of chloride concentration on the salt-fog corrosion of several Mg-Al alloys (AZ31, AZ91) has been reported by Merino et al. [14]. As the alloy’s Al percentage decreased and the temperature and Cl concentration increased, the corrosion attack intensified. The susceptibility of RE-containing wrought alloys WE43-T6 and WE54-T6 to microgalvanic corrosion has been investigated with scanning Kelvin probe force microscopy by Coy et al. [15]. The examination revealed the presence of different types of precipitates located within the matrix and at the grain boundaries, with a composition corresponding to Mg_12_(RE, Y), sub-micron spherical Zr-rich and cubic Y-rich precipitates, and needle-like b-phase precipitates.

The effect of grain refinement, through friction stir processing, on the corrosion behavior of wrought Mg alloys was described by Argade et al. 2012 [16]. Contrasting the peak aged and processed samples with the friction stir processed samples revealed enhanced pitting potential. The corrosion resistance of WE43 alloy, after hot extrusion, has been evaluated by Przondziono et al. [17]. The results of the tests showed that the corrosion resistance of the extruded magnesium alloy WE43 decreases as the molar concentration of the NaCl solution increases.

Taking into account the above research, the present work is a comparative study, regarding AZ31, AZ61, and RE-containing WE43 wrought Mg alloys, focusing on the microstructural characterization of salt fog corrosion. The study includes measurements of mass loss and depth of attack, and sheds light on the characterization of corrosion products.

## 2. Materials and Methods

Hot rolled Mg-Al-Zn magnesium alloys, AZ31 and AZ61, as well as a RE-containing alloy, WE43-T5, in the form of thin plates with a nominal thickness of 2.0 mm were used for the present study [18]. The chemical composition of the alloys is given in Table 1. The materials are characterized as high-purity, as the concentration of the contaminants (Fe, Ni, and Cu) was held under certain limits, since it is known that these elements have a detrimental effect on the corrosion resistance of Mg alloys [19]. After the rolling procedure, the materials were subjected to annealing. The AZ31 was treated for 30 min at 300 °C, the AZ61 for 1 h at 250 °C and the WE43-T5 for 16 h at 200 °C, as mentioned in Table 2.

**Figure 1:**
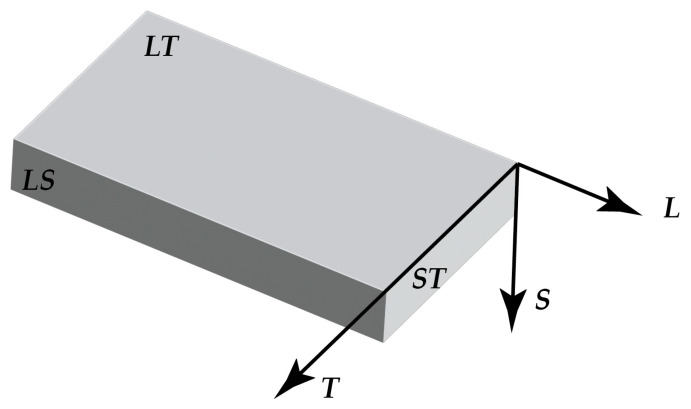
Schematic representation of plane and direction designation of sheet material.

The microstructure of the as–received AZ31, AZ61, and WE43-T5 alloy sheets was revealed by light optical examination of metallographic sections, in the (LS), (LT), and (ST) planes with respect to longitudinal-rolling L, the transverse T, and the thickness S directions of the rolled sheets, as indicated in Figure 1. Standard cutting, grinding, and polishing techniques for Mg-alloys were applied. Metallographic specimen preparation consisted of cutting the specimens with an abrasive cutting wheel Struers Accutom (Struers, Ballerup, Denmark), embedding them in epoxy resin, and grinding them with consecutively finer SiC papers (500–4000 grit). The specimens were subsequently polished down to 1 μm using polycrystalline diamond suspension (Struers, Ballerup, Denmark); then down to 0.045μm using alumina suspension. Etching of AZ31 and AZ61 alloys was performed using a solution, consisting of 5 mL acetic acid, 6 gr picric acid, 10 mL H_2_O, and 70 mL ethanol. Alloy WE43-T5 was etched with the picral etchant (5% picric acid in ethanol). Transmission Electron Microscopy analysis was performed with a JEOL 2000FX (JEOL, Tokyo, Japan), operating at 200 kV microscope equipped with a NORAN EDX system. The specimens studied in TEM were ion-beam thinned and electro polished. An electrolyte with a composition of 20% perchloric acid, 5% 2-butoxyetanol, and 75% ethanol (by volume) was used. The samples were rinsed in methanol and ethanol after etching. Some specimens were also ion milled with a 5 keV double argon beam for a few minutes after electropolishing, when preferential etching was observed along grain boundaries. In the TEM, the samples were tilted to various orientations where the position of the grain boundaries could be clearly distinguished. For the SEM analysis, a FEI Quanta 200F equipped with an EDAX EDX system was used, operating at 20 kV. Microhardness testing was performed using a Vickers microhardness intender and a load of 0.1 Kg.

Salt fog corrosion testing was performed according to specification ASTM B 117 [20]. The dimensions of the exposed specimens were 125 mm × 75 mm × 2 mm, the longer dimension being parallel to the rolling direction of the sheet. Prior to corrosion exposure, the surfaces of the specimens were cleaned by immersion in a 10 vol.% HNO_3_ solution, followed by immersion in a 10 wt% NaOH solution. During immersion in each solution, the surfaces of the specimens were gently brushed with a soft brush. After cleaning, the specimens were dried and weighed, to determine the initial mass of each specimen. Each specimen was subsequently exposed to the test solution for a specified period between 1 and 48 h. Following corrosion exposure, the specimens were removed from the salt spray chamber and were subjected to repetitive cleaning and weighing cycles, according to specification ASTM G 1 [21]. After cleaning and weighing, the specimens were subjected to macroscopic optical examination to determine the depth of attack. Measurement of the depth of attack was performed in the optical microscope, by measuring the difference of focusing levels at the bottom and at the surface of the corrosion pits according to specification ASTM G 46 [22]. For each specimen, a total of 30 randomly selected pits on the exposed surface were measured, to produce a statistically credible measurement.

XPS analysis of the corroded surfaces of AZ61 and WE43-T5 was performed on a Kratos Axis Ultra^DLD^ spectrometer using monochromatic Al Kα radiation (hν = 1486.6 eV). The chamber pressure was 1 × 10^−8^ Torr. The analysis was performed using an acceleration voltage 15 kV and emission current 10 mA.

## 3. Results

### 3.1. Microstructural Characterization of Uncorroded Materials

**Figure 2:**
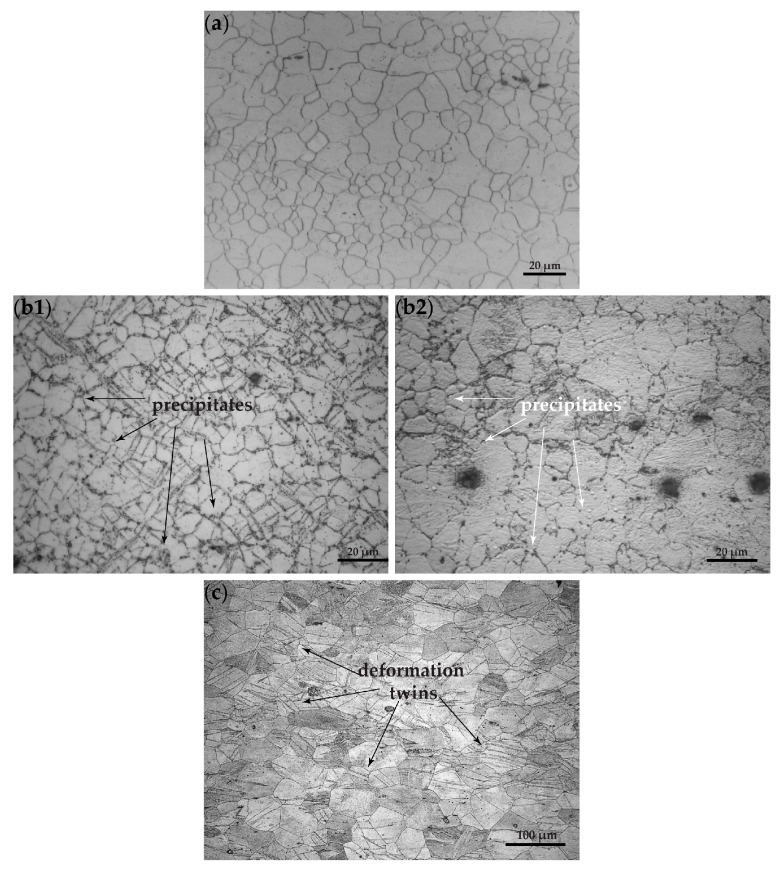
Optical microscopy micrographs depicting the microstructure of magnesium alloys in the (LS) plane: (**a**) AZ31; (**b1**) AZ61 top area; (**b2**) AZ61 middle area; and (**c**) WE43-T5.

Figure 2a depicts the microstructure of the AZ31 alloy sheet in the LS plane, consisting of recrystallized equiaxed grains of the hcp Mg α-phase. The microstructure of AZ31 mainly contains precipitates in the grain interior, as also shown by TEM analysis in Section 3.1.1. Microhardness measurements were performed on the three planes. The LT plane exhibits the lowest microhardness, 48 ± 3 HV, while the material at the LS and ST planes exhibits corresponding microhardness values of 58 ± 3.5 HV and 55 ± 3.1 HV (Table 3). Figure 2b depicts the microstructure of the AZ61 alloy sheet in the LS plane. A recrystallized equiaxed structure is observed, as in the case of AZ31, with no evidence of annealing twins. In AZ61, some precipitates are revealed along grain boundaries, as indicated in Figure 2b1,b2. TEM investigation of the AZ61 alloy (reported in Section 3.1.2 below) identified these precipitates as belonging to the β-phase, Mg_17_Al_12_. All three planes of the alloy exhibit similar microhardness values (LT-63 ± 3.2 HV, LS-60 ± 3.5 HV, and ST-60 ± 4 HV). The noted exception is in the LS plane, the area near the rolling surface of the specimen, which appears to have an increased microhardness of 74 ± 2.5 HV, and its microstructure reveals twinning, probably superficially induced during the forming of the material sheet (Figure 2b1). It should be mentioned that the microstructure of the AZ61 alloy is not as homogeneous as that of AZ31; precipitates were observed mainly at grain boundaries. 

Figure 2c depicts the microstructure of the WE43-T5 alloy sheet in the LS plane. A heavily deformed structure is observed, as indicated by the large number of deformation twins. The microhardness values obtained for this alloy (Table 3) are increased with the LT and LS planes higher than the ST plane (LT-91.5 ± 4.5 HV, LS-87.2 ± 3.5 HV, and ST-81.8 ± 2.9 HV). 

Regarding grain size, WE-43 exhibits a larger grain size than AZ31 and AZ61, as well as significant scatter. For the LT plane, the grain size was measured within the range 20–80 μm, for the LS plane within 20–50 μm, and for the ST plane within 30–60 μm. Special mention should be made of the considerably large grain size, exhibiting an average value of 60 μm. In comparison, for the AZ31 alloy, the grain size was measured at 13 μm for the LT, at 12 μm for the LS plane, and at 18 μm for the ST plane. Similar values were measured for the alloy AZ61, at 16 μm for the LT plane, at 11 μm for the LS plane near the surface, corresponding with the higher value observed in the microhardness measurements, and at 15 μm in the central area of the plane. Finally, the grain size for the ST plane was measured at 18 μm.

#### 3.1.1. TEM of Alloy AZ31

**Figure 3:**
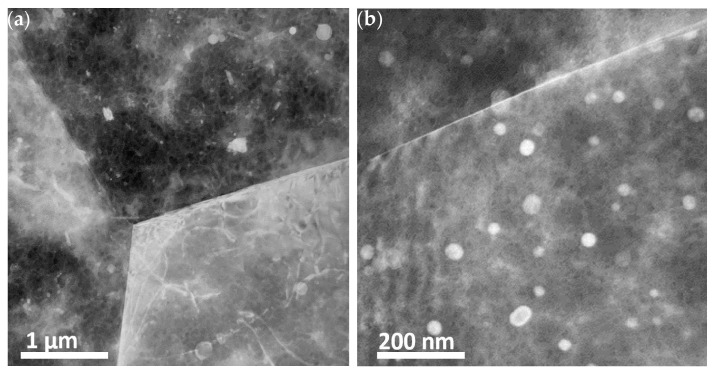
TEM images of alloy AZ31 (**a**) bright field (BF) overview and (**b**) higher magnification showing a random distribution of particles of various sizes ranging up to a few nanometers in diameter.

**Figure 4:**
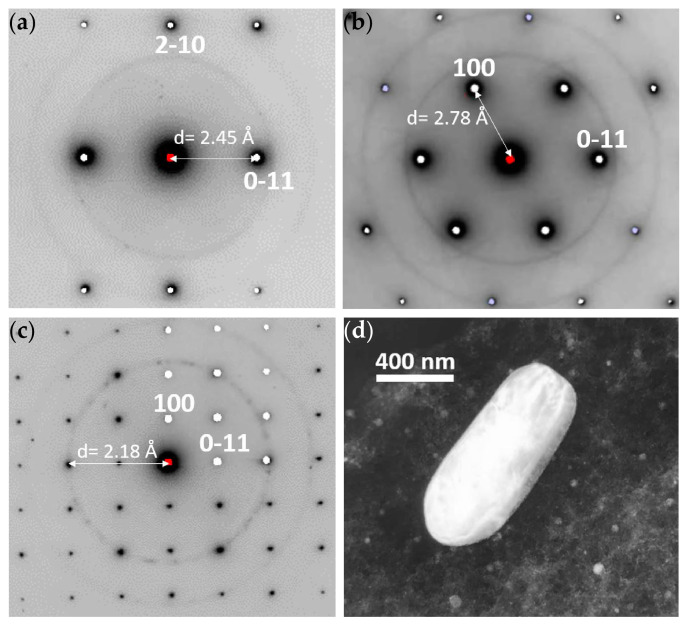
SAD images with overlaid simulated patterns of Mg depicted in the (**a**) [122] and (**b**) [011] ZA orientations; (**c**) SAD from the Al_11_Mn_5_ particle depicted in (**d**) with confirming simulated pattern overlaid. The ring patterns in (**a**–**c**) are found to be consistent with MgO-precipitates.

In the bright field (BF) image in Figure 3a, three grains of magnesium alloy AZ31 can be seen. No evidence for localized/preferred precipitation along the grain boundaries was found. SAD images with overlaid simulated patterns were recorded for both grains and grain boundaries, as shown in Figure 4a for the [122] zone axis and in Figure 4b for the [011] zone axis. The diffraction patterns from the matrix were indexed in agreement with hcp Mg. In addition to larger precipitates (also observed with SEM as discussed below), smaller precipitates were found randomly dispersed in the microstructure of the samples. The random distribution of the precipitates/small particles, mainly in the grain interior, are more clearly seen in Figure 3b, where the sample was tilted so that the Mg grains do not strongly scatter. Figure 4c depicts a SAD pattern from a Al_11_Mn_5_ particle depicted in Figure 4d with the confirming simulated pattern overlaid. The ring patterns in Figure 4a to c are found to be consistent with MgO-precipitates. The presence of MgO particles embedded in the alloy microstructure is attributed to oxidation occurring upon material processing (hot rolling). 

#### 3.1.2. TEM of Alloy AZ61

**Figure 5:**
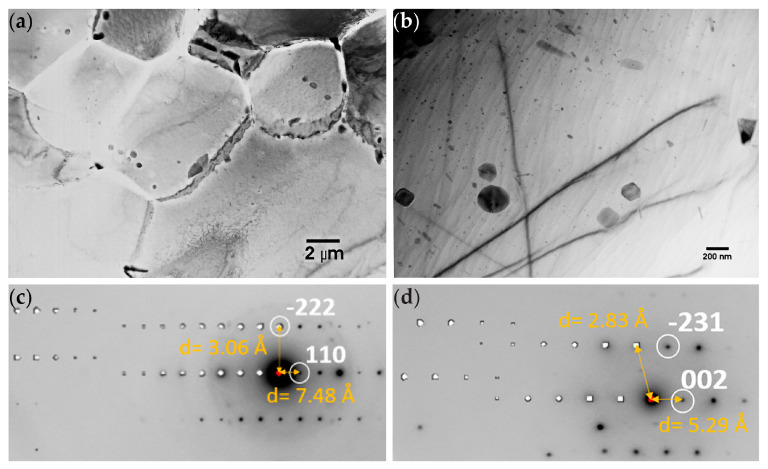
TEM images of alloy AZ61: (**a**) Grains with clearly etched grain boundaries; (**b**) Particles of the secondary phase identified as Mg_17_Al_12_ inside a Mg grain; (**c**) [1−12] and (**d**) [320] SAD ZA patterns with corresponding simulated patterns indexed in agreement with Mg_17_Al_12_.

**Figure 6:**
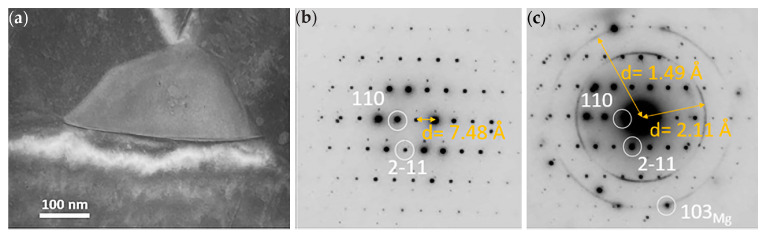
TEM images of alloy AZ61: (**a**) TEM micrograph of a Mg_17_Al_12_ particle located on a triple point grain boundary with (**b**) corresponding [1−11] SAD pattern and (**c**) pattern including contributions from MgO (ring pattern) and Mg matrix (additional reflections).

The TEM images in Figure 5a show grains of several microns in diameter. Particles of a secondary phase were found both along the grain boundaries as well as inside the grains, as depicted in Figure 5b. Energy dispersive x-ray analysis (EDS) of the particles showed that they were rich in Al and Mg (~2:3 ratio) and contained a few atomic % Zn. Selected area diffraction (SAD) patterns, depicted in Figure 5c,d from the secondary phase, were indexed in agreement with Mg_17_Al_12_ β-phase, which has previously been reported in the literature to have space group I-43m and lattice parameter 10.54 Å [23]. TEM images of a Mg_17_Al_12_ particle located on a triple point grain boundary are presented in Figure 6a. Corresponding [1,2,3,4,5,6,7,8,9,10,11] SAD patterns, including contributions from MgO (ring pattern) and Mg matrix (additional reflections), are depicted in Figure 6b,c.

#### 3.1.3. TEM of Alloy WE43-T5 

**Figure 7:**
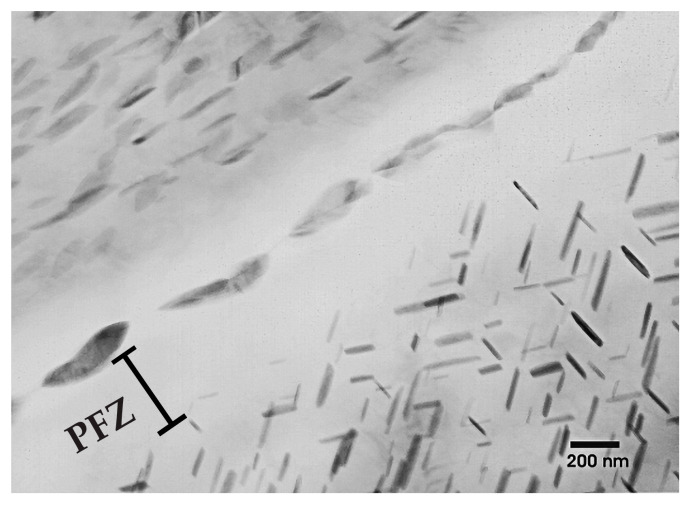
Grain boundary in the WE43-T5 alloy showing a PFZ and precipitates in the matrix and along the grain boundary.

**Figure 8:**
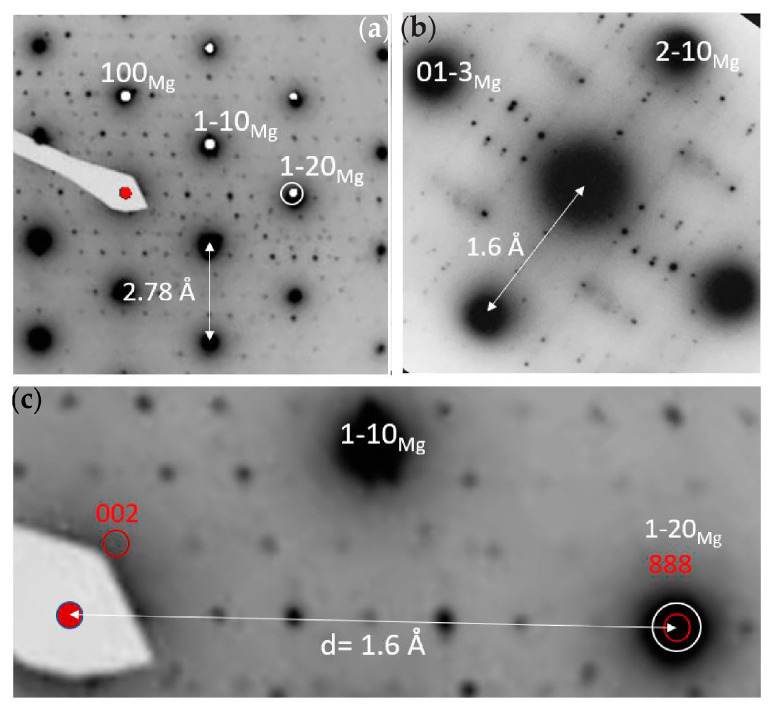
SAD patterns of β precipitate in alloy WE43-T5 showing: (**a**) [001]Mg//[1−10]β ZA patterns with corresponding [001]Mg simulation; (**b**) SAD pattern and simulation of the [362]Mg ZA with contributions from plural equivalent orientations of the β precipitates in the Mg matrix; (**c**) (1–20)Mg //(111)β orientation relationship and consistent with face-centered cubic β precipitates with a~ 22 Å cell dimension.

The WE43-T5 alloy contains plate-shaped precipitates, as shown in Figure 7. The precipitates are found both in the interior of the grains as well as along the grain boundaries. Near the grain boundaries, there is a 300 nm wide precipitation free zone (PFZ), which indicates strong diffusion towards the grain boundaries. EDS analysis of the precipitates, depicted in Table 4, shows that they are enriched in Nd and Y as compared to the surrounding Mg matrix. EDS analysis showed a Nd:Y ratio equal to 5:3 for the precipitates formed along the grain boundaries, as shown in Table 4, and a ratio of 1:1 for the precipitates present in the matrix. SAD patterns of the magnesium matrix containing plate-shaped precipitates are shown in Figure 8. The patterns were indexed in agreement with the phase referred to as the β phase in the literature [24,25,26], having a FCC structure with lattice constant 22.2 Å. 

### 3.2. Microstructural Features after Salt Fog Corrosion

#### 3.2.1. Macro Examination

**Figure 9:**
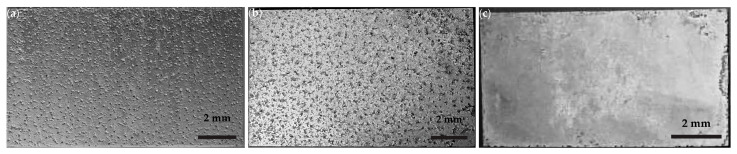
Optical macrographs of surface exposed to the salt–spray test for 24 h: (**a**) alloy AZ31; (**b**) alloy AZ61; and (**c**) WE43-T5.

The optical macrographs of Figure 9a–c depict the exposed surfaces of AZ31, AZ61, and WE43-T5 (LT surface) alloy specimens tested for 24 h. The AZ61 alloy surface appears to be more severely attacked. WE43-T5 appears to be more resistant to corrosion as the surface is almost unaffected.

#### 3.2.2. Metallographic Sections

**Figure 10:**
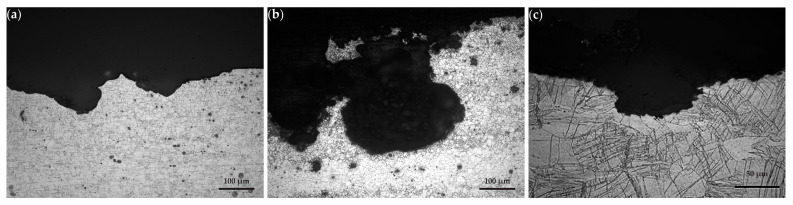
Typical metallographic sections in the longitudinal (L) directions of: (**a**) AZ31 specimen tested for 6 h; (**b**) AZ61 specimen tested also for 6 h; and (**c**) WE43-T5 specimen tested for 24 h.

Light optical micrographs of sections corresponding to the (LS) plane are shown in Figure 10a, Figure 10b, and Figure 10c for AZ31, AZ61, and WE43-T5, respectively. In the case of AZ31 and AZ61, corrosion proceeds by preferential dissolution of the α phase (hcp-Mg). Corrosion attack is more severe for AZ61. Alloy WE43-T5 exhibits a higher corrosion resistance overall, as it is characterized by isolated pitting. 

#### 3.2.3. Depth of Attack and Relative Mass Loss

**Figure 11:**
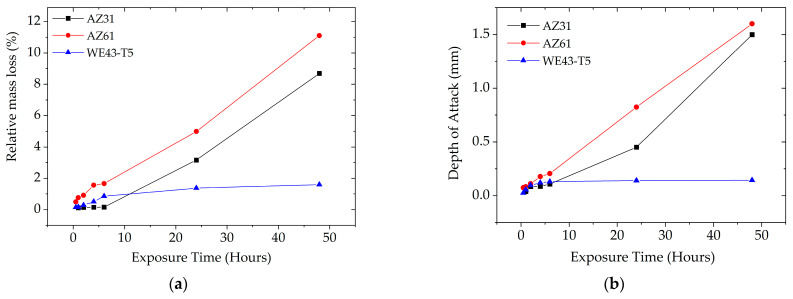
Comparison of: (**a**) relative mass loss and (**b**) maximum measured depth of attack of AZ31, AZ61, and WE43-T5 specimens as functions of exposure time.

The depth of attack and relative mass loss were determined as a function of exposure time and are depicted in Figure 11. These parameters vary almost linearly with exposure time for AZ31 and AZ61, with the latter exhibiting higher values, in agreement with the microscopic observations discussed above. The maximum depth of attack for AZ61 reaches the value of 1.5 mm at 48 h of exposure, which is to be compared with the 2 mm thickness of the sheet. Regarding WE43-T5, the depth of attack and relative mass loss varies almost linearly with exposure time for the first 4 h of exposure. Then the rate of mass loss reaches a plateau from 6 to 48 h of exposure, where there is almost no increase in mass loss and depth of attack.

### 3.3. Analysis of Corrosion Products

**Figure 12:**
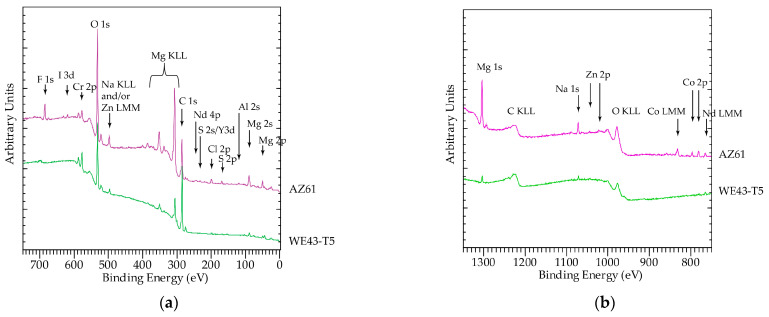
Survey spectra of samples analyzed: (**a**) 0–700 (eV) and (**b**) 800–1300 (eV).

**Figure 13:**
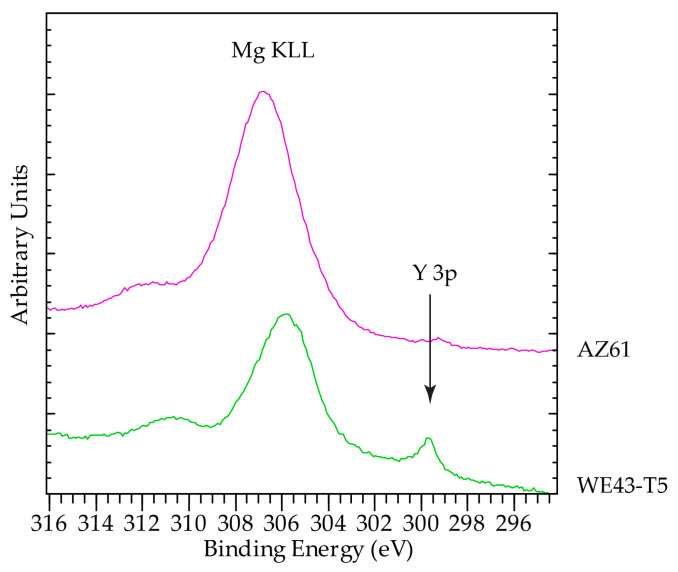
Mg KLL and Y 3p high resolution scans.

XPS analysis of corroded surfaces was performed on the alloys that exhibited the best and worst corrosion behaviors, namely the AZ61 and WE43-T5 after 24 h of exposure in the salt fog environment. Survey spectra are shown in Figure 12. In addition to the standard expected elements C, O, and Mg, the surfaces of the samples analyzed contained significant amount of Cr in most probably Cr(OH)_2_ form. Cr is due to the cleaning process. The presence of Na and Cl was also found to be more enhanced on the surfaces of AZ61. Sample AZ61 also contained Al and Zn Co, I, and F. The corroded surface of WE43-T5 contained Y. It is difficult to be certain about the exact oxidation state of Y, because Y 3d overlaps with S 2s as S presence of S was also detected. As shown in Figure 13, the position of Y 3p (299.7 eV) is higher than the value reported in the literature for pure Y (299.3 eV), and therefore we suggest that Y is in an oxidized form. 

**Figure 14:**
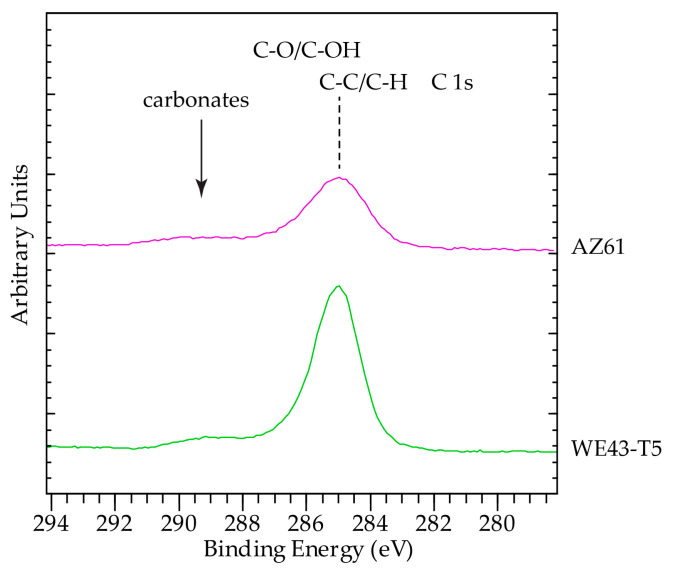
C 1s peak. The spectra were calibrated for charging by setting the C 1s peak maximum at 285 eV.

**Figure 15:**
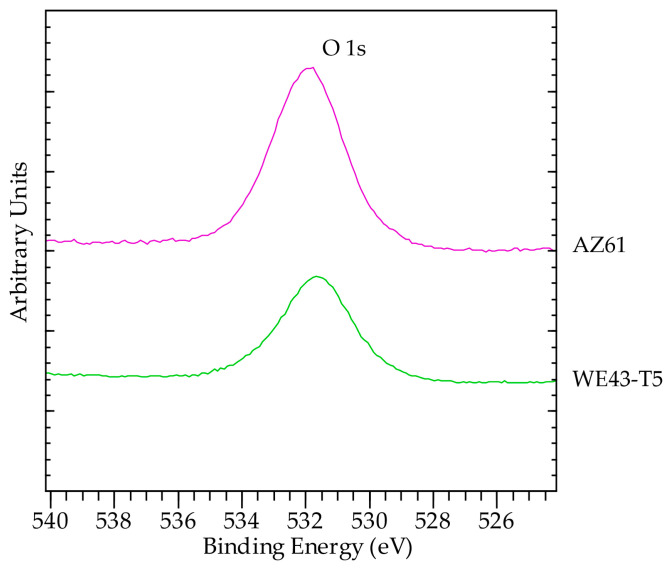
High resolution O1s scans. The peak position corresponds to OH^-^ and/or adsorbed species.

The surface of WE43-T5 contained a higher amount of C, as compared to sample AZ61, implying a higher amount of surface contamination for the corroded WE43-T5 as compared to the AZ61 (Figure 14). This is evident by the increase in the post C1s peak background slope. The increased amount of C masks the signals from O (Figure 15) and Mg (Figure 13), which are more intense in the sample with less C, i.e., AZ61. Regarding the chemical state of Mg, unlike many other elements present in the surface of the samples, Mg has the big advantage of being the base element, and therefore, the spectroscopic features were intense. In addition, with the employed excitation radiation (Al Kα), we can acquire all known Mg peaks. The latter offers the advantage of utilizing the Auger parameter concept mentioned above. This is very beneficial, since a comparison of absolute peak positions is problematic due to energy referencing issues. In this study, the Mg 2p-KLL and 1s-KLL Auger parameters (AP) were measured to irrespectively identify the chemical state to charging or energy referencing effects. The Auger parameter is measured by adding the binding energy (BE) of the photoelectron ejected from energy level i (i = 1s or 2p in the Mg case) to the kinetic energy (KE) of the equivalent emitted Auger electron ijj (ijj = the KLL Auger transition in the Mg case). The measured values together with the literature values are shown in Table 5. Both 1s-*KLL* and 2p-*KLL* Auger parameters for Mg show that Mg is present as MgO/Mg(OH)_2_ in both AZ61 and WE43-T5 alloys.

## 4. Discussion

### 4.1. Corroded AZ31 and AZ61 Alloys

Al and Zn were present on the corroded surface of AZ61. These two elements have been previously reported to be incorporated in the surface oxide film of AZ91 alloy [27] and to promote hydrogen bonding in the hydroxide film [28]. In the absence of impurities in binary alloys, Baliga et al. [29] and Baliga and Tsakiropoulos [30,31,32] attributed the improved corrosion resistance of splat-quenched Mg-Al alloys with Al > 10 wt% to the incorporation of Al ions in the prior oxide/hydroxide film, and the subsequent formation of a corrosion film consisting of a double layered hydroxycarbonate/chloride brucite structure. Lunder et al. [33] showed that the Mg_17_Al_12_ phase existing in the microstructure of AZ91 is corrosion resistant in 5 wt% NaCl solution for a pH range, which is wider than the range for testing both Mg and Al. The reason for the passivating behavior of this phase is the co-existence of Al and Mg oxide in both corroded and non-corroded conditions. It was suggested that when Mg_17_Al_12_ is either finely dispersed (via rapid solidification processing-ennoblement of the Mg matrix) or continuously precipitated along grain boundaries by heat treatment, it could enhance corrosion resistance. 

In the above context, two main conclusions can be drawn. The presence of Al in the oxide/hydroxide surface layer is not sufficient to form a passivated protective layer on the surface of both AZ alloys, owing to the low Al content in the base alloy. The better corrosion performance of the AZ31 alloy is attributed to the finer distribution of the nobler than the Mg-matrix precipitates (Al-Mn and/or Mg-Al phases) throughout the alloy microstructure and the absence of second phases-precipitates at grain boundaries. The latter prevents intergranular corrosion taking place. The localisation of the precipitates along the grain boundaries and the subsequent micro-galvanic effects with the adjacent Mg matrix, in combination with the high grain boundary area (due to microstructure refinement), seem to be responsible for extensive intergranular corrosion of the AZ61 alloy. We suggest that this microstructural feature in combination with the ineffective participation of Al in the oxide/hydroxide film are responsible for the inferior corrosion performance of this alloy.

### 4.2. Corroded WE43-T5 Alloy

Incorporation of Y has been suggested to take place in the surface oxide film of WE54 [27], with the presence of Y in the oxide being enhanced by a factor of 5. Corrosion of Mg-10 wt% Gd, Mg-10 wt% Dy, Mg-3 wt% Nd-0.4 wt% Zr, Mg-10 wt% Gd-3 wt% Nd-0.3 wt% Zr, Mg-10 wt% Dy-3 wt% Nd-0.4 wt% Zr, and WE54 in 5 wt% NaCl saturated with Mg(OH)_2_ indicated the presence of heavy rare-earth elements enhanced by the corrosion resistance, although the open circuit potential was shifted to more negative values [34,35]. The improved corrosion performance was attributed to passivation due to the formation of MgH_2_, as well as to the presence of Gd, Nd, and Dy compounds in the MgO/Mg(OH)_2_ film of corroded Mg-Gd(Dy)-Nd-Zr alloys [36]. It has been suggested that the beneficial effect of the rare earth additions arises from the stabilization of MgO, Mg(OH)_2_, and 3MgCO_3_.Mg(OH)_2_.3H_2_O in the surfaces of these alloys.

Research on the effect of rare earth additions [37,38] in rapidly solidified Mg alloys (up to 20 wt% Nd, 26 wt% Y, and 21 wt% Ce) attributed the improved corrosion resistance of these alloys to the surface enrichment in RE element, and to the formation of second phase particles containing rare earth elements (e.g., the β-Mg_25_Y_4_). These particles have slightly more negative electrode potential, they offer cathodic protection, and act as anodic sites, thus sacrificially protecting the Mg matrix. The degradation rate of WE43-T5 due to corrosion, as expressed by relative mass loss and depth of attack as a function of corrosion exposure time in Figure 11, is much lower than the degradation rate of AZ31 and AZ61. The superior corrosion performance of the WE43 alloy as compared to the AZ31 and AZ61 alloys is attributed to the incorporation of Y in the oxide/hydroxide film, and to the cathodic protection offered by the relatively uniformly distributed Y- and Nd- containing particles. These particles consist of elements having electronegativity values lower but very close to Mg, in contrast to Al/Mn, which are main constituents of the ppts in the microstructure of the AZ alloys. It is thus expected that the ppts in the WE43 alloys have slightly more negative electrode potential and act as anodic sites, sacrificially protecting the Mg majority phase as explained above. In contrast, the difference between the electrode potentials of the electropositive Mg matrix and the more electronegative Al/Mn containing precipitates in the AZ alloys is larger than that the WE43 alloys, and thus the corrosion resistance of the former is lower than the latter. 

Table 5 shows two sets of Auger parameters (AP) for the surface oxide, the 2p-KLL and the 1s-KLL. The former is more “bulk” sensitive as compared to the latter since the Mg 2p photoelectrons escape from a larger depth compared to the Mg 1s ones. The Mg 2p-KLL AP for the AZ61 is slightly lower than that for both WE43-T5 and the literature value for MgO, whilst the opposite is seen for the Mg 1s-KLL, which is higher for the AZ61 compared to the WE43-T5. The AP is interpreted as a measurement of the core hole screening efficiency [39,40]. The lower “bulk” and higher “surface” AP for the AZ61 and the higher “bulk” and lower “surface” AP for the WE43-T5 correspond to the surface enrichment in Al/Zn and Y/Nd, respectively. Al and Zn have more delocalized/mobile available valence electrons (s, p-type) as compared to Y and Nd, whose available valence electrons are more localized (4d and 4f, respectively). The increased screening of the surface oxide can be attributed to the availability of the extra mobile valence electrons provided by the Al/Zn sites incorporated in the Mg oxide. Similarly, the reduced screening of the surface oxide in the WE43-T5 is attributed to the fact that the available extra electrons from the Y/Nd sites are localized and cannot provide effective screening. Although the differences are small and close to the experimental error (see Table 5), the increased/reduced core hole screening in the surface layer may be considered as a reflection of the increased/reduced electric conductivity. It could therefore suggest that the incorporation of Y/Nd increases the insulating character of the surface oxide, thus providing an additional explanation for the higher corrosion resistance of the WE43-T5 alloy as compared to the AZ31 and AZ61.

## 5. Conclusions

From the results presented above, the following conclusions can be drawn:

All three alloys exhibited a recrystallized equiaxed structure after hot rolling with twinning in the AZ61 and WE43-T5 alloys, while twins were absent in the AZ31 alloy.

TEM analysis did not reveal grain boundary precipitates in the AZ31 alloy. However, the AZ61 alloy exhibited the presence of precipitates along the grain boundaries and in the grain interior. In the WE43-T5 alloy, plate-like precipitates were observed on grain boundaries and the grain interior with associated PFZs. These precipitates were enriched in Nd and Y, and were identified as FCC β phase. 

Regarding corrosion, the WE43-T5 alloy exhibited a better corrosion resistance than AZ31 and AZ61 under salt fog testing, indicated by the lower depth of attack and lower weight loss. 

The second phases in the microstructure determined the corrosion resistance of AZ31 and AZ61. The second phases in the AZ31 and AZ61 alloys (based on Al-Mg and Al-Mn phases) were nobler than the Mg matrix and catholically acted, thus sacrificing the Mg matrix. The superior corrosion performance of the WE43-T5 alloy as compared to the AZ31 and AZ61 alloys was attributed to the incorporation of Y in the oxide/hydroxide film, and to the cathodic protection offered by the relatively uniformly distributed Y- and Nd-containing particles, which were less noble than the Mg-matrix.

## Figures and Tables

**Table 1:** Chemical composition of alloys AZ31, AZ61, and WE43-T5 (in %mass).

	Al	Zn	Mn	Fe	Ni	Cu	Si	Mg
**AZ31**	3.06	0.80	0.25	0.003	<0.001	0.001	-	bal.
**AZ61**	6	0.72	0.33	0.003	≤0.001	≤0.01	<0.01	bal.
								
**WE43-T5**	**Yttrium**			**Neodymium**		**Zirconium**		**Mg**
	3.7–4.3			2.4–4.4%		0.4%		bal

**Table 2:** Heat treatment of alloys AZ31, AZ61, and WE43-T5.

	Temperature (°C)	Time
**AZ31**	300	30 min
**AZ61**	250	60 min
**WE43-T5**	200	16 h

**Table 3:** Average grain size and microhardness of alloys AZ31, AZ61, and WE43-T5.

	Plane	Average Grain Size (μm)	Microhardness (HV)
**AZ31**	**LT**	13	48 ± 3.5
	**LS**	12	58 ± 3.5
	**ST**	18	55 ± 4
**AZ61**	**LT**	16	63 ± 3.2
	**LS**	13	60 ± 3.5
	**ST**	18	60 ± 4
**WE43-T5**	**LT**	50	91.5 ± 4.5
	**LS**	35	87.2 ± 3.5
	**ST**	45	81.8 ± 2.9

**Table 4:** EDS result from precipitates on a grain boundary and from precipitates in the matrix of alloy WE43-T5. The Cu signal originates from the sample holder.

	Precipitates on a Grain Boundary				Precipitates in the Matrix			
Element Line	Net Counts	Net Counts Error	Atom %	Atom % Error	Net Counts	Net Counts Error	Atom %	Atom % Error
**Mg K**	10,319	+/−143	89.10	+/−1.23	24,488	+/−205	94.65	+/−0.79
**Cu K**	1056	+/−61	2.49	+/−0.14	1409	+/−68	1.49	+/−0.07
**Cu L**	4	+/−38	---	---	0	+/−30	---	---
**Y K**	974	+/−88	3.35	+/−0.30	1216	+/−129	1.87	+/−0.20
**Y L**	1388	+/−113	---	---	1807	+/−117	---	---
**Y M**	0	+/−48	---	---	0	+/−72	---	---
**Nd L**	3297	+/−143	5.05	+/−0.22	2903	+/−141	1.99	+/−0.10
**Nd M**	132	+/−54	---	---	124	+/−58	---	---
**Total**			100.00				100.00	

**Table 5:** Mg 2p-KLL and Mg 1s-KLL final state Auger parameters in corroded surfaces.

	AZ61	WE43-T5	Mg (Literature)	MgO (Literature)	Mg(OH)_2_ (Literature)
**BE of Mg 2p (eV)**	50.2 ± 0.1	49.9 ± 0.1	49.4–49.95	50.25–51.1	49.5
**BE of Mg 1s (eV)**	1304.45 ± 0.1	1303.9 ± 0.1	1303–1303.8	1303.9	1302.4
**KE of Mg *KLL* (eV)**	1179.8 ± 0.2	1180.8 ± 0.1	1184.9–1185.9	1181.3	
**2p-*KLL* AP (eV)**	1230 ± 0.22	1230.7 ± 0.22	1234.7–1235.45	1230.8–1231.6	
**1s-*KLL* AP**	2485.25 ± 0.22	2484.7 ± 0.22	2488.3–2489.3	2485–2485.2	

## Data Availability

Not applicable.
